# Design of neuro-swarming computational solver for the fractional Bagley–Torvik mathematical model

**DOI:** 10.1140/epjp/s13360-022-02421-3

**Published:** 2022-02-18

**Authors:** Juan L. G. Guirao, Zulqurnain Sabir, Muhammad Asif Zahoor Raja, Dumitru Baleanu

**Affiliations:** 1 Department of Applied Mathematics and Statistics, Technical University of Cartagena, Hospital de Marina, 30203 Cartagena, Spain; 2 Department of Mathematics, Faculty of Science, King Abdulaziz University, P.O. Box 80203, Jeddah, 21589 Saudi Arabia; 3 Lab Theor Cosmology, Int Centre of Gravity and Cosmos, TUSUR, Tomsk, Russia 634050; 4 Department of Mathematics and Statistics, Hazara University, Mansehra, Pakistan; 5 Future Technology Research Center, National Yunlin University of Science and Technology, 123 University Road, Section 3, Douliou, Yunlin 64002 Taiwan, Republic of China; 6 Department of Mathematics, Cankaya University, Ankara, Turkey; 7 Institute of Space Science, Magurele-Bucharest, Romania

## Abstract

This study is to introduce a novel design and implementation of a neuro-swarming computational numerical procedure for numerical treatment of the fractional Bagley–Torvik mathematical model (FBTMM). The optimization procedures based on the global search with particle swarm optimization (PSO) and local search via active-set approach (ASA), while Mayer wavelet kernel-based activation function used in neural network (MWNNs) modeling, i.e., MWNN-PSOASA, to solve the FBTMM. The efficiency of the proposed stochastic solver MWNN-GAASA is utilized to solve three different variants based on the fractional order of the FBTMM. For the meticulousness of the stochastic solver MWNN-PSOASA, the obtained and exact solutions are compared for each variant of the FBTMM with reasonable accuracy. For the reliability of the stochastic solver MWNN-PSOASA, the statistical investigations are provided based on the stability, robustness, accuracy and convergence metrics.

## Introduction

The fractional Bagley–Torvik mathematical model (FBTMM) has achieved the huge attention of the research community in recent years. The fractional kinds of derivatives represent the physical network dynamics, a rigid plate based on the Newtonian fluid, and the frequency- dependent systems of the damping properties [1–4]. The numerical, approximate and analytical form of the FBTMM has been performed by many scientists and reported in [6–10]. While few other utmost deterministic and stochastic numerical schemes [11–19] are listed in Table 1 in terms of novel methodology exploited for the solutions, publication year, and necessary remarks to highlight their significance in the reported literature for FBTMM.

**Table 1:** A brief literature review of numerical solver for FBTMM

Index	Method	Remarks
[11] in 1998	Podlubny’s consecutive approximation	Novel numerical solution
[12] in 2002	Deterministic numerical scheme	Convergence established
[13] in 2007	Differential transform method	Novel numerical solver
[14] in 2008	Adomian decomposition method	Novel analytical solution
[15] in 2008	He’s variational iteration method	Viable analytic method
[16] in 2009	Matrix approach of discretization	Novel discretization
[17] in 2010	Shooting collocation approach	Efficient scheme
[18] in 2010	Taylor collocation method	Power series approach
[19] in 2011	Genetic algorithms and neural networks	Novel stochastic solver
[20] in 2011	Neural networks and Swarm intelligence	Viable stochastic solver
[20] in 2012	Haar wavelets operational matrix	Novel wavelets approach
[22] in 2017	Sequential quadratic programing	Fractional neural network
[23] in 2020	Interior-point method	Fluid dynamics problem
[24] in 2020	Galerkin approximations	Numerical scheme
[25] in 2020	Exponential spline approximation	Novel spline method
[26] in 2020	Jacobi collocation methods	Power series approach
[27] in 2021	Generalized Bessel polynomial	Power series method
[28] in 2021	Quadratic finite element mentod	Numerical computing
[29] in 2021	Lie symmetry analysis method	Numerical analysis

The present study is to solve the FBTMM by using a competent soft computing approach based on a Mayer wavelet neural network (MWNN) using the optimization procedures of particle swarm optimization (PSO) along with active-set algorithm (ASA), i.e., MWNN-PSOASA. The general form of the FBTMM is provided as [20–23]:

1 $$ \left\{ \begin{gathered} a_{1} \frac{{d^{2} v\left( \tau \right)}}{{d\tau^{2} }} + a_{2} \frac{{d^{\alpha } v\left( \tau \right)}}{{d\tau^{\alpha } }} + a_{3} v\left( \tau \right) = h\left( \tau \right), \hfill \\ \frac{{d^{\beta } v\left( \tau \right)}}{{d\tau^{\beta } }} = a_{\beta } ,\beta = 0,\,\,1, \hfill \\ \end{gathered} \right. $$

where $a_{\alpha }$ indicates the initial conditions, $\lambda$ represents the derivative based on the fractional-order with 1.25, 1.5, and 1.75, $v(\tau )$ is the solution of above Eq. (1), while $a_{1}$, $a_{2}$, and $a_{3}$ are the constant values The FBTMM represented in Eq. (1) was the pioneering work of Bagley and Torvik introducing on the motion of an absorbed plate using the Newtonian fluid [3].

### Problem statement

The stochastic computing solvers have been generally applied to the singular, nonlinear and dynamical systems based on the platform of neural network together with the swarming/ evolutionary optimization schemes [30–32]. The stochastic solvers have been applied in diverse applications, few of them are coronavirus SITR model [33, 34], singular doubly differential systems [35], fluid dynamics problems [36], HIV infections modeling systems [37, 38], and electric circuits model [39, 40]. The authors are motivated by keeping these stochastic-based applications to design a computing solver for the FBTMM. Therefore, the objective of study is to introduce a novel design and implementation of a neuro-swarming computational numerical procedure MWNN-PSOASA for numerical treatment of the fractional Bagley–Torvik mathematical model (FBTMM) by exploiting global search optimization procedures via particle swarm optimization (PSO) and local search via active-set approach (ASA), while Mayer wavelet kernel-based activation function used in neural network (MWNNs) modeling.

### Novelty and inspiration

The novelty and significance of the research investigations are briefly described in this section. The literature review presented for FBTMM, one can decipher evidently that a large variant of deterministic solvers have been introduced by research community for solving the FBTMM while few studies of stochastic solvers are available for finding the approximate solutions for FBTMM, Therefore a novel fractional neural network is presented for the solution of FBTMM by exploiting the strength of fractional Mayer wavelet neural networks (MWNN) based modeling of the fractional derivative terms in ODE (1) and training of these networks are performed by hybrid heuristics having global search with PSO and ASA based local refinements, i.e., MWNN-PSOASA. The designed solver MWNN-PSOASA is used efficiently and effectively to solve the FBTMM numerically. The achieved form of the numerical results is compared with the accessible true/exact solutions, which shows the precision, reliability, constancy, and convergence of the designed solver MWNN-PSOASA. The reliability of the results based on the designed solver MWNN-PSOASA is further presented using the statistical procedures of Mean, semi interquartile range (S.I.R), Minimum (Min), standard deviation (STD), Theil's inequality coefficient (TIC), mean square error (MSE) and Maximum (Max). Besides, the accurate and reasonably stable outcomes of the FBTMM through the designed solver MWNN-PSOASA, robustness, smooth processes, and exhaustive pertinence are other significant perks of the scheme.

### Organization

The organization of this study is considered as: The methodology of MWNN-PSOASA is accessible in Sect. 2. The performance operators are shown in Sect. 3. The comprehensive results detail is given in Section 4. Final remarks and upcoming research directions are given in the last section.

## Methodology

**Fig. 1:**
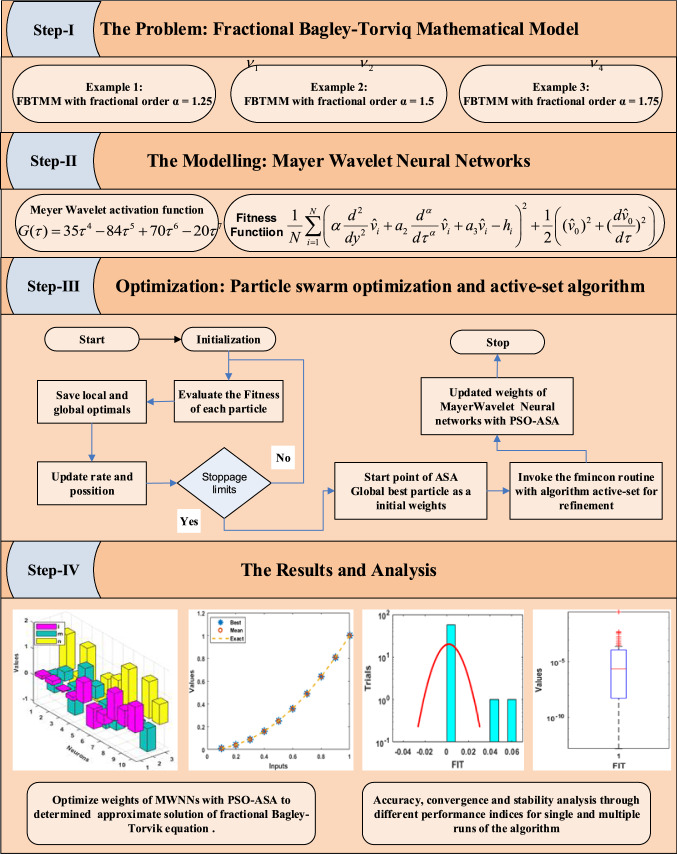
Workflow diagram of MWNN-PSOASA for solving fractional Bagley-Torvik equation

This section represents the methodology of the designed solver by using the Mayer wavelet neural network along with the optimization of PSOASA to solve the FBTMM. The genetic flow diagram of proposed MWNN-PSOASA for solving FBTMM is provided in Fig. 1, in which the process blocks in four steps are presented. The construction of the FBTMM, merit function based on the mean square error, and the PSOASA optimization is also presented in this section.

### Objective function: MWNN

In this section, the FBTMM solutions are signified by $\hat{v}(\tau )$, whereas, $D^{(n)} \hat{v}(\tau )$ and $D^{\alpha } \hat{v}(\tau )$ provides the integer derivatives of order *n* and fractional form of the derivative. The mathematical formulations of these systems by means of continuous mapping in neural networks models are given as:

2 $$ \begin{gathered} \hat{v}\left( \tau \right) = \sum\limits_{i\, = \,1}^{m} {l_{i} G(m_{i} \tau + n_{i} )} \hfill \\ \frac{{d^{(n)} }}{{dy^{(n)} }}\hat{v}\left( \tau \right) = \sum\limits_{i = 1}^{m} {l_{i} \frac{{d^{(n)} }}{{dy^{(n)} }}G(m_{i} \tau + n_{i} )} \hfill \\ \frac{{d^{\alpha } }}{{dy^{\alpha } }}\hat{v}\left( \tau \right) = \sum\limits_{i = 1}^{m} {l_{i} \frac{{d^{\alpha } }}{{d\tau^{\alpha } }}G(m_{i} \tau + n_{i} )} \hfill \\ \end{gathered} $$

where, neurons are represented by m, while ***l***, **m,** and **n** indicate the weights of the weight vector (**W)** represented as:

$${\varvec{W}} = [{\varvec{l}},{\varvec{m}},{\varvec{n}}], \; \rm{for}\;{\varvec{l}} = [l_{1} ,l_{2} ,...,l_{m} ],\,\,{\varvec{m}} = [m_{1} ,m_{2} ,...,m_{m} ]\,\,and\,\,{\varvec{n}} = [n_{1} ,n_{2} ,...,n_{m} ]$$

An objective function-based Mayer wavelet is written as:

3 $$ G\left( \tau \right) = 35\tau^{4} - 84\tau^{5} + 70\tau^{6} - 20\tau^{7} $$

The updated Eq. (2) using the above values is become as:

$$ \hat{v}\left( \tau \right) = \sum\limits_{i = 1}^{m} {l_{i} \left( \begin{gathered} 35(m_{i} \tau + n_{i} )^{4} - 84(m_{i} \tau + n_{i} )^{5} + \hfill \\ 70(m_{i} \tau + n_{i} )^{6} - 20(m_{i} \tau n_{i} )^{7} \hfill \\ \end{gathered} \right)} , $$

4 $$ \frac{{d^{(n)} }}{{d\tau^{(n)} }}\hat{v}\left( \tau \right) = \sum\limits_{i = 1}^{m} {l_{i} \left( \begin{gathered} 35\frac{{d^{(n)} }}{{d\tau^{(n)} }}(m_{i} \tau + n_{i} )^{4} - 84\frac{{d^{(n)} }}{{d\tau^{(n)} }}(m_{i} \tau + n_{i} )^{5} + \hfill \\ 70\frac{{d^{(n)} }}{{d\tau^{(n)} }}(m_{i} \tau + n_{i} )^{6} - 20\frac{{d^{(n)} }}{{d\tau^{(n)} }}(m_{i} \tau + n_{i} )^{7} \hfill \\ \end{gathered} \right)} , $$

$$ \frac{{d^{\alpha } }}{{d\tau^{\alpha } }}\hat{v}\left( \tau \right) = \sum\limits_{i = 1}^{m} {l_{i} \left( \begin{gathered} 35\frac{{d^{\alpha } }}{{d\tau^{\alpha } }}(m_{i} \tau + n_{i} )^{4} - 84\frac{{d^{\alpha } }}{{d\tau^{\alpha } }}(m_{i} \tau + n_{i} )^{5} + \hfill \\ 70\frac{{d^{\alpha } }}{{d\tau^{\alpha } }}(m_{i} \tau + n_{i} )^{6} - 20\frac{{d^{\alpha } }}{{d\tau^{\alpha } }}(m_{i} \tau + n_{i} )^{7} \hfill \\ \end{gathered} \right)} . $$

The combination of the MWNN with the optimization of PSOASA is used to solve the FBTMM based on the availability of appropriate **W** values. For the ANN weights, an objective function $e_{F}$ is given as:

5 $$ e_{F} = e_{F - 1} + e_{F - 2} $$

Here $e_{F - 1}$* and *$e_{F - 2}$ are the objective functions based on the FBTMM and the ICs of Eq. (1), respectively written as:

6 $$ e_{F - 1} = \frac{1}{N}\sum\limits_{i = 1}^{N} {\left( {\alpha \frac{{d^{2} }}{{dy^{2} }}\hat{v}_{i} + a_{2} \frac{{d^{\alpha } }}{{d\tau^{\alpha } }}\hat{v}_{i} + a_{3} \hat{v}_{i} - h_{i} } \right)}^{{2}} , $$

7 $$ e_{F - 2} = \frac{1}{2}\left( {(\hat{v}_{0} )^{2} + (\frac{{d\hat{v}_{0} }}{d\tau })^{2} } \right) $$

*for* $$Nh = 1,\,\,\,\hat{v}_{i} = \hat{v}\left( {\tau_{i} } \right),\,\,h_{i} = h\left( {\tau_{i} } \right)\,,\,\,\,\tau_{i} = ih$$*.*

$$ \frac{1}{N}\sum\limits_{i = 1}^{N} {\left( {\alpha \frac{{d^{2} }}{{dy^{2} }}\hat{v}_{i} + a_{2} \frac{{d^{\alpha } }}{{d\tau^{\alpha } }}\hat{v}_{i} + a_{3} \hat{v}_{i} - h_{i} } \right)}^{{2}} + \frac{1}{2}\left( {(\hat{v}_{0} )^{2} + (\frac{{d\hat{v}_{0} }}{d\tau })^{2} } \right) $$

### Networks optimization: PSOASA

The parameter optimization, i.e., weights, for the MWNN models are obtained using the hybridization of computing procedures of particle swarm intelligence PSO as an efficacious global search aided with active set algorithms (ASAs) for efficient local refinement mechanism to solve the variants of FBTMM in equation (1).

Particle swarm optimization is a computational swarm intelligence approach, which is used to optimize a model through the process of iteration to improve the applicant outcomes, i.e., candidate solutions of a specific optimization tasks, with respect to assume quality measures and constraints. The PSO normally solves a model by using the population of applicant outcomes called swarm and each candidate solution is represented by the particles. The PSO algorithms operate with the adjustment of these particles during each flight in search-space based on the mathematical representations of the particles velocity and position in terms of previous velocity, inertia weight of velocity, cognitive learning block via local best particle, and social learning mechanism via global search particle. The movement of the particles is affected by its local prominent based positions; however, it is also directed to the best-recognized positions, which are efficient as improved positions of other particles. This is projected to transfer the swarm to the best results. Additional necessary elaborative details, underlying theory, mathematical representation, scope, and applications in diversified fields can be seen in [41–43] and references mentioned in them. In recent decades, PSO is implemented to plant diseases diagnosis and prediction [44], nonlinear Bratu systems governing the fuel ignition model [45], identification of control autoregressive moving average systems [46], reactive power planning [47], and thermal cloaking and shielding devices [48].

In order to control and speed up the convergence performance of the global search PSO, the optimization through the hybridization with local search method is implemented for speedy adjustment of the parameters. The active-set algorithm is one of the quick, rapid, and efficient local search schemes, which is famous to find the optimal performances in different fields. ASA is an effectual convex optimization tool that is implemented for unconstrained and constrained systems. Few prominent applications of utmost performance of the ASA include embedded system of predictive control [49], pressure-dependent network of water supply systems [50], local decay of residuals in dual gradient method [51], nonlinear singular heat conduction model [52], and warehouse location problem [53]. Therefore, in the presented study, a memetic computing paradigm PSOASA based on global search efficacy of PSO aided with speedy tuning of parameter with ASA are exploited for finding the known adjustable of MWNN models for solving the FBTMM in Eq. (1).

## Results and discussions

In this section, the solutions of three different variants based on the FBTMM are provided by using the integrated design heuristics of MWNN-PSOASA. The precision and convergence on the basis of sixty number of autonomous trails MWNN-PSOASA are presented using sufficient large number of graphical and numerical illustrations with elaborative details for solving the variants of the FBTMM. While the information/outcomes of different performance indices for the analysis of proposed MWNN-PSOASA are given in this section.

The mathematical form of the performance gages of TIC and MSE are presented to solve the FBTMM as follows:

8 $$ {\text{TIC = }}\frac{{\sqrt {\frac{1}{n}\sum\nolimits_{m = 1}^{n} {\left( {v_{m} - \hat{v}_{m} } \right)}^{2} } }}{{\left( {\sqrt {\frac{1}{n}\sum\nolimits_{m = 1}^{n} {\hat{v}_{m}^{2} } } + \sqrt {\frac{1}{n}\sum\nolimits_{m = 1}^{n} {\hat{v}_{m}^{2} } } } \right)}}, $$

9 $$ {\text{MSE = }}\sum\limits_{i = 1}^{k} {\left( {v_{m} - \hat{v}_{m} } \right)}^{2} , $$

where *n* is total number of grid points, i.e., *τ*_*m*_, *m* = 1, 2, …, *n*, while $v_{m}$ is the reference solution for *m*^*th*^ grid point while $\hat{v}_{m}$ is proposed approximate solution for the *m*^*th*^ grid point.

### *Example 1*

Consider an FBTMM given in Eq. (1) using the values of $h(\tau ) = \tau^{2} + \frac{\Gamma (3)}{{\Gamma (1.75)}}\tau^{0.75} + 2$, $\alpha = 1.25$ and $a_{1} = a_{2} = a_{3} = 1$ is given as:

10 $$ \left\{ \begin{gathered} \frac{{d^{2} v}}{{d\tau^{2} }} + \frac{{d^{1.25} v}}{{d\tau^{1.25} }} + v(\tau ) = \tau^{2} + \frac{\Gamma (3)}{{\Gamma (1.75)}}\tau^{0.75} + 2,\;0 \le \tau \le 1, \hfill \\ v(0) = 0,\,\,\frac{dv(0)}{{d\tau }} = 0. \hfill \\ \end{gathered} \right. $$

An error function is derived as:

11 $$ \begin{aligned} e_{F} &= \frac{1}{N}\sum\limits_{i = 1}^{N} {\left( {\frac{{d^{2} }}{{d\tau^{2} }}\hat{v}_{i} + \frac{{d^{1.25} }}{{d\tau^{1.25} }}\hat{v}_{i} + \hat{v}_{i} - \tau^{2} - \frac{\Gamma (3)}{{\Gamma (1.75)}}\tau^{0.75} - 2} \right)}^{{2}}\\ &\quad + \frac{1}{2}\sum\limits_{i = 1}^{m} {\left( {(\hat{v}_{0} )^{2} + (\frac{{d\hat{v}_{0} }}{d\tau })^{2} } \right)} , \end{aligned} $$

### *Example 2*

Consider an FBTMM given in Eq. (1) using the values of $h(\tau ) = \tau^{2} + 4\sqrt {\frac{\tau }{\pi }} + 2$, $\alpha = 1.5$ and $a_{1} = a_{2} = a_{3} = 1$ is given as:

12 $$ \left\{ \begin{gathered} \frac{{d^{2} v}}{{d\tau^{2} }} + \frac{{d^{1.5} v}}{{d\tau^{1.5} }} + v(\tau ) = \tau^{2} + 4\sqrt {\frac{\tau }{\pi }} + 2,\;0 \le \tau \le 1, \hfill \\ v(0) = 0,\,\,\frac{dv(0)}{{d\tau }} = 0. \hfill \\ \end{gathered} \right. $$

An error function is derived as:

13 $$ \begin{aligned} E_{F}& =  \frac{1}{N}\sum\limits_{i = 1}^{N} {\left( {\frac{{d^{2} }}{{dy^{2} }}\hat{u}_{i} + \frac{{d^{1.5} }}{{dy^{1.5} }}\hat{u}_{i} + \hat{u}_{i} - y^{2} - 4\sqrt {\frac{y}{\pi }} - 2} \right)}^{{2}} \\ &\qquad+  \frac{1}{2}\sum\limits_{i = 1}^{m} {\left( {(\hat{u}_{0} )^{2} + \left( {\frac{{d\hat{u}_{0} }}{dy}} \right)^{2} } \right)} , \\ \end{aligned} $$

### *Example 3*

Consider an FBTMM given in Eq. (1) using the values of $h(\tau ) = \tau^{2} + \frac{\Gamma (3)}{{\Gamma (1.25)}}\hat{v}(\tau )^{0.25} + 2$, $\alpha = 1.75$ and $a_{1} = a_{2} = a_{3} = 1$ is written as:

14 $$ \left\{ \begin{gathered} \frac{{d^{2} v}}{{d\tau^{2} }} + \frac{{d^{1.75} v}}{{d\tau^{1.75} }} + v(\tau ) = \tau^{2} + \frac{\Gamma (3)}{{\Gamma (1.25)}}\tau^{0.25} + 2,\;0 \le \tau \le 1, \hfill \\ v(0) = 0,\,\,\frac{dv(0)}{{d\tau }} = 0. \hfill \\ \end{gathered} \right. $$

An error function is derived as:

15 $$ \begin{aligned} e_{F} &= \frac{1}{N}\sum\limits_{i = 1}^{N} {\left( {\frac{{d^{2} }}{{d\tau^{2} }}\hat{v}_{i} + \frac{{d^{1.75} }}{{d\tau^{1.75} }}\hat{v}_{i} + \hat{v}_{i} - \tau^{2} - \frac{\Gamma (3)}{{\Gamma (1.25)}}\tau^{0.25} - 2} \right)}^{{2}} \\ &\quad + \frac{1}{2}\sum\limits_{i = 1}^{m} {\left( {(\hat{v}_{0} )^{2} + (\frac{{d\hat{v}_{0} }}{d\tau })^{2} } \right)} , \end{aligned} $$

The exact solutions of the FBTMM are $v(\tau ) = \tau^{2}$.

The numerical performances of each example based on the FBTMM are observed using the hybridization of local and global search capabilities of PSOASA. The optimization procedures are applied for sixty independent runs of MWNN-PSOASA to form a larger dataset for better analysis of solution dynamics of FBTMM. The accomplished/adjusted weights of MWNNs are used to obtained numerical solutions of the FBTMM and necessary comparison with reference/available exact solution is conducted to assess the proposed solutions. The obtained mathematical results through the MWNN-PSOASA for each example of the FBTMM are expressed in mathematical form as follows:

16 $$ \begin{aligned} \hat{v}_{E - 1} &= 0.1250\left( {35( - 0.675\tau + 1.4050)^{4} - 84( - 0.675\tau + 1.4050)^{5} + 70( - 0.675\tau + 1.4050)^{6} - 20( - 0.675\tau + 1.4050)^{7} } \right)\\ & \quad+ {0}{\text{.1565}}\left( {35(0.3447\tau - {0}{\text{.4161}})^{4} - 84(0.3447\tau - {0}{\text{.4161}})^{5} + 70(0.3447\tau - {0}{\text{.4161}})^{6} - 20(0.3447\tau - {0}{\text{.4161}})^{7} } \right)\\ & \quad + {0}{\text{.0352}}\left( {35( - 1.082\tau + 1.3359)^{4} - 84( - 1.08\tau + 1.3359)^{5} + 70( - 1.082\tau + 1.3359)^{6} - 20( - 1.082\tau + 1.3359)^{7} } \right)\\ & \quad - 0.1419\left( {35({0}{\text{.9416}}\tau - 0.4897)^{4} - 84({0}{\text{.941}}\tau - 0.4897)^{5} + 70({0}{\text{.941}}\tau - 0.4897)^{6} - 20({0}{\text{.9416}}\tau - 0.4897)^{7} } \right)\\ & \quad  + {0}{\text{.7821}}\left( {35(0.4722\tau + 0.5975)^{4} - 84(0.4722\tau + 0.5975)^{5} + 70(0.4722\tau + 0.5975)^{6} - 20(0.4722\tau + 0.5975)^{7} } \right)\\ & \quad - 0.7659\left( {35( - 0.914\tau + {0}{\text{.7870}})^{4} - 84( - 0.914\tau + {0}{\text{.787}})^{5} + 70( - 0.914\tau + {0}{\text{.7870}})^{6} - 20( - 0.914\tau + {0}{\text{.7870}})^{7} } \right)\\ & \quad - 0.3828\left( {35( - 0.751\tau + 1.4290)^{4} - 84( - 0.751\tau + 1.4290)^{5} + 70( - 0.751\tau + 1.4290)^{6} - 20( - 0.751\tau + 1.4290)^{7} } \right)\\ & \quad + 1.0566\left( {35( - 0.167\tau - 0.1258)^{4} - 84( - 0.167\tau - 0.125)^{5} + 70( - 0.167\tau - 0.1258)^{6} - 20( - 0.167\tau - 0.1258)^{7} } \right)\\ & \quad + {0}{\text{.6174}}\left( {35( - 0.071\tau + 1.4174)^{4} - 84( - 0.071\tau + 1.4174)^{5} + 70( - 0.071\tau + 1.4174)^{6} - 20( - 0.071\tau + 1.4174)^{7} } \right)\\ & \quad + 0.9066\left( {35( - {0}{\text{.83}}\tau + 0.7723)^{4} - 84( - {0}{\text{.836}}\tau + 0.7723)^{5} + 70( - {0}{\text{.836}}\tau + 0.7723)^{6} - 20( - {0}{\text{.836}}\tau + 0.7723)^{7} } \right),  \end{aligned} $$

17 $$ \begin{aligned} \hat{v}_{E - 2} &= 0.1060\left( {35( - 1.387\tau + 1.1953)^{4} - 84( - 1.387\tau + 1.1953)^{5} + 70( - 1.387\tau + 1.1953)^{6} - 20( - 1.387\tau + 1.1953)^{7} } \right)\\ & \quad  - 1.8006\left( {35( - 0.3968\tau - {0}{\text{.1376}})^{4} - 84( - 0.396\tau - {0}{\text{.1376}})^{5} + 70( - 0.396\tau - {0}{\text{.1376}})^{6} - 20( - 0.39\tau - {0}{\text{.1376}})^{7} } \right)\\ & \quad + {0}{\text{.0330}}\left( {35( - 0.4350\tau + 1.1337)^{4} - 84( - 0.435\tau + 1.1337)^{5} + 70( - 0.435\tau + 1.1337)^{6} - 20( - 0.435\tau + 1.1337)^{7} } \right) \\ & \quad  + 1.0266\left( {35({0}{\text{.7659}}\tau - 0.2077)^{4} - 84({0}{\text{.7659}}\tau - 0.207)^{5} + 70({0}{\text{.7659}}\tau - 0.2077)^{6} - 20({0}{\text{.7659}}\tau - 0.2077)^{7} } \right)\\ & \quad - 0.0092\left( {35( - 0.826\tau + 1.4202)^{4} - 84( - 0.826\tau + 1.4202)^{5} + 70( - 0.826\tau + 1.4202)^{6} - 20( - 0.826\tau + 1.4202)^{7} } \right) \\ & \quad - 0.2724\left( {35({0}{\text{.5454}}\tau + 1.2393)^{4} - 84({0}{\text{.5454}}\tau + 1.2393)^{5} + 70({0}{\text{.5454}}\tau + 1.2393)^{6} - 20({0}{\text{.5454}}\tau + 1.2393)^{7} } \right) \\ & \quad + {0}{\text{.1652}}\left( {35( - 0.526\tau + 1.2138)^{4} - 84( - 0.526\tau + 1.2138)^{5} + 70( - 0.526\tau + 1.2138)^{6} - 20( - 0.526\tau + 1.2138)^{7} } \right) \\ & \quad  - 0.3594\left( {35( - 0.013\tau - 0.2252)^{4} - 84( - 0.013\tau - 0.2252)^{5} + 70( - 0.013\tau - 0.2252)^{6} - 20( - 0.013\tau - 0.2252)^{7} } \right) \\ & \quad - 1.0433\left( {35({0}{\text{.6590}}\tau - 0.2011)^{4} - 84({0}{\text{.6590}}\tau - 0.2011)^{5} + 70({0}{\text{.6590}}\tau - 0.2011)^{6} - 20({0}{\text{.6590}}\tau - 0.2011)^{7} } \right) \\ & \quad  + ... + 0.2031\left( {35(1.2456\tau - 0.0762)^{4} - 84(1.2456\tau - 0.0762)^{5} + 70(1.2456\tau - 0.0762)^{6} - 20(1.2456\tau - 0.0762)^{7} } \right), \end{aligned} $$

18 $$ \begin{aligned}   \hat{v}_{{E - 3}}  &  =  - 12.8773\left( {35(0.0783\tau  - 0.0847)^{4}  - 84(0.0783\tau  - 0.0847)^{5}  + 70(0.0783\tau  - 0.0847)^{6}  - 20(0.078\tau  - 0.0847)^{7} } \right) \\     & \quad  + 1.0608\left( {35( - 0.2304\tau  + 0.4748)^{4}  - 84( - 0.230\tau  + 0.4748)^{5}  + 70( - 0.230\tau  + 0.4748)^{6}  - 20( - 0.230\tau  + 0.4748)^{7} } \right) \\     & \quad  - 0.4913\left( {35({\text{0}}{\text{.1076}}\tau  - 0.4530)^{4}  - 84({\text{0}}{\text{.1076}}\tau  - 0.4530)^{5}  + 70({\text{0}}{\text{.1076}}\tau  - 0.4530)^{6}  - 20({\text{0}}{\text{.1076}}\tau  - 0.4530)^{7} } \right) \\     & \quad  + {\text{0}}{\text{.9486}}\left( {35( - 0.0310\tau  + 0.0305)^{4}  - 84( - 0.031\tau  + 0.0305)^{5}  + 70( - 0.031\tau  + 0.0305)^{6}  - 20( - 0.031\tau  + 0.0305)^{7} } \right) \\     & \quad  - 0.0171\left( {35( - 0.3685\tau  + 1.7255)^{4}  - 84( - 0.368\tau  + 1.7255)^{5}  + 70( - 0.368\tau  + 1.7255)^{6}  - 20( - 0.368\tau  + 1.7255)^{7} } \right) \\     & \quad  - 1.1819\left( {35({\text{0}}{\text{.0629}}\tau  - 0.1260)^{4}  - 84({\text{0}}{\text{.0629}}\tau  - 0.1260)^{5}  + 70({\text{0}}{\text{.0629}}\tau  - 0.1260)^{6}  - 20({\text{0}}{\text{.0629}}\tau  - 0.126)^{7} } \right) \\     & \quad  + 1.5776\left( {35({\text{0}}{\text{.4436}}\tau {\text{ + 0}}{\text{.1399}})^{4}  - 84({\text{0}}{\text{.4436}}\tau {\text{ + 0}}{\text{.1399}})^{5}  + 70({\text{0}}{\text{.4436}}\tau {\text{ + 0}}{\text{.1399}})^{6}  - 20({\text{0}}{\text{.44361}}\tau {\text{ + 0}}{\text{.1399}})^{7} } \right) \\     & \quad  + {\text{0}}{\text{.6170}}\left( {35( - 0.979\tau  + 1.0302)^{4}  - 84( - 0.979\tau  + 1.0302)^{5}  + 70( - 0.979\tau  + 1.0302)^{6}  - 20( - 0.979\tau  + 1.0302)^{7} } \right) \\     & \quad  + {\text{0}}{\text{.1524}}\left( {35( - 0.898\tau  + 0.7207)^{4}  - 84( - 0.898\tau  + 0.7207)^{5}  + 70( - 0.898\tau  + 0.7207)^{6}  - 20( - 0.898\tau  + 0.7207)^{7} } \right) \\     & \quad  + ... + 0.3625\left( {35(1.0742\tau  - 0.0409)^{4}  - 84(1.0742\tau  - 0.0409)^{5}  + 70(1.0742\tau  - 0.0409)^{6}  - 20(1.0742\tau  - 0.0409)^{7} } \right), \\  \end{aligned}  $$

**Fig. 2:**
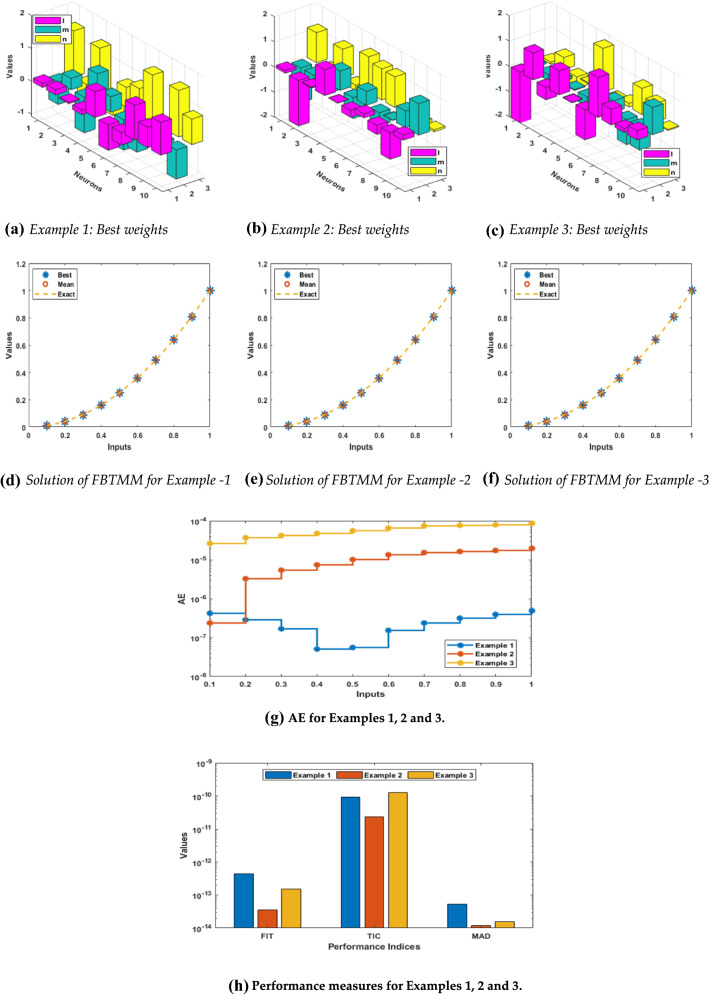
Results, (**a**)-(**c**) Best weights, (**d**)-(**f**) AE, (**g**) and performance operators (**h**) for solving the FBTMM

The graphs represented in Fig. 2, i.e., subfigures (a), (b) and (c), show the numerical values of the weights of MWNNs obtained from proposed integrated swarm intelligence method MWNN-PSOASA for FBTMM based examples 1 2 and 3, respectively. Fig. 1, subfigures (d), (e), and (f) represents the overlapping of the results based on the best, worst and mean solutions for each example of the FBTMM. This perfectly matching of the outcomes indicate the precision and exactness of the MWNN-PSOASA for solving FBTMM. The plots of the absolute error (AE) are drawn in Fig 1g for each example of the FBTMM. It is noticed for example 1 that 70% AE values lie around 10^−06^ to 10^−07^ and 30% AE values are calculated around 10^−07^ to 10^−08^. In 2nd example, 90% AE values lie around 10^−05^ to 10^−06^ and 10% AE values lie around 10^−06^ to 10^−07^. While for 3rd example the AE values are calculated around 10^-04^ to 10^−05^. On the behalf of these best ranges of the AE values, one can conclude that the designed scheme MWNN-PSOASA is an accurate and precise. The performances detail based on the Fitness (FIT), TIC, and MSE for each example of the FBTMM is drawn in Fig. 1h. The FIT standards lie around 10^−12^ to 10^−13^, 10^−13^ to 10^−14^_,_ and 10^−12^ to 10^−13^ for examples 1, 2, and 3, respectively. The TIC standards are calculated around 10^−10^ to 10^−11^ to solve each example of the FBTMM. While the MSE standards are calculated around 10^-13^ to 10^-14^ to solve each example of the FBTMM.

**Fig. 3:**
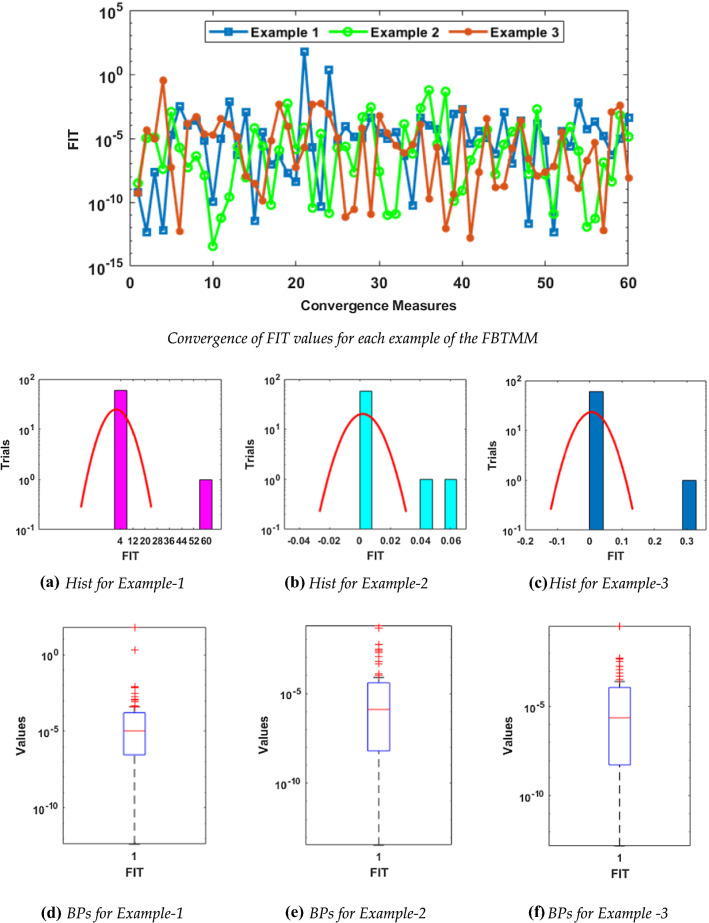
Convergence of FIT values for each example of the FBTMM with Hist and BPs using 10 neurons

**Fig. 4:**
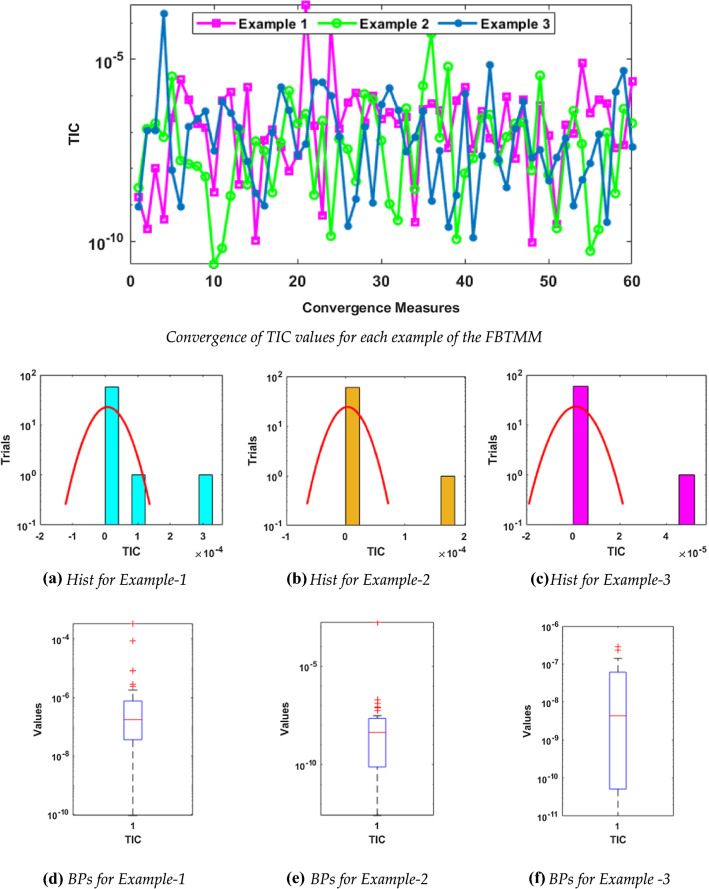
Convergence of TIC values for each example of the FBTMM with Hist and BPs using 10 neurons

**Fig. 5:**
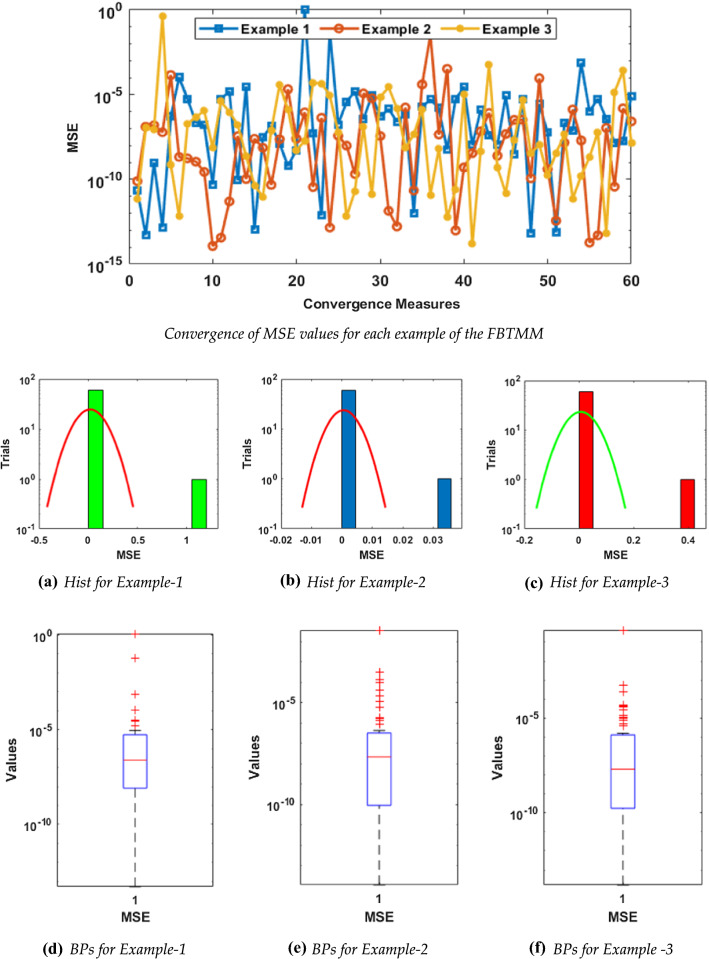
Convergence of MSE values for each example of the FBTMM with Hist and BPs using 10 neurons

The statistical performance for the FIT, TIC, and MSE is conducted via the boxplots (BPs) and histograms (Hist) studies and outcomes are portrayed in Figs. 3, 4, and 5 for all three examples in case of FIT, TIC, and MSE, respectively. Figure 3 illustrates the FIT values for each example of the FBTMM. It is illustrated in these figures that the FIT, TIC, and MSE measures are found around 10^−04^ to 10^−12^, 10^−06^_,_ to 10^−10^ and 10^−04^ to 10^−10^ for each example of the FBTMM, respectively. One can conclude from these outcomes that around 75% independent trials of MWNN-PSOASA achieved precise level of the accuracy.

In order to authenticate the accuracy, precision, convergence, and efficiency analysis, the statistical results in terms of Min, median (Med), Mean, STD, Max, and S.I.R operators are tabulated in Table 2 which are calculated for sixty independent runs using the FMWNN-PSOASA to solve the FBTMM. The Min and Max values indicate the best and worst runs, respectively, while S.I.R performances are the 0.5 times difference of 3^rd^ and 1^st^ quartiles. The effective and small magnitudes of Min, S.I.R, Med, STD and Max indicate the constancy and precision of the proposed integrated heuristics of MWNN-PSOASA to solve the variants of the FBTMM in examples 1, 2, and 3. For the convergence analysis using the statistical operators based on the FIT, TIC, and MSE with different set of magnitude are calculated for multiple execution of MWNN-PSOASA and results are tabulated in Table 3 for all three examples of FBTMM. The sufficient large number of independent execution of MWNN-PSOASA achieved the FIT, TIC and MSE less than 10^−04,^ that prove the worth of design scheme for solving FBTMM.

**Table 2:** Statistics operators via FMWNN-PSOASA to solve each example of the FBTMM

	Mode	Solutions of FBTMM using different statistical measures									
		0.1	0.2	0.3	0.4	0.5	0.6	0.7	0.8	0.9	1
Example-1	Min	1 × 10^–07^	3 × 10^–07^	2 × 10^–07^	5 × 10^–08^	6 × 10^–08^	2 × 10^–07^	2 × 10^–07^	2 × 10^–07^	2 × 10^–07^	1 × 10^–07^
	Mean	3 × 10^–02^	2 × 10^–02^	1 × 10^–02^	6 × 10–03	1 × 10^–02^	2 × 10^–02^	3 × 10^–02^	3 × 10^–02^	4 × 10^–02^	4 × 10^–02^
	Max	2 × 10^–01^	1 × 10^–01^	6 × 10^–01^	2 × 10^–01^	3 × 10^–01^	8 × 10^–01^	1 × 10^–01^	1 × 10^–01^	2 × 10^–01^	2 × 10^–01^
	Med	1 × 10^–04^	3 × 10^–04^	4 × 10^–04^	4 × 10^–04^	4 × 10^–04^	5 × 10^–04^	6 × 10^–04^	8 × 10^–04^	8 × 10^–04^	8 × 10^–04^
	SIR	3 × 10^–04^	6 × 10^–04^	8 × 10^–04^	9 × 10^–04^	1 × 10^–03^	1 × 10^–03^	1 × 10^–03^	1 × 10^–03^	1 × 10^–03^	1 × 10^–03^
	STD	2 × 10^–01^	1 × 10^–01^	8 × 10^–02^	3 × 10^–02^	5 × 10^–02^	1 × 10^–01^	1 × 10^–01^	2 × 10^–01^	2 × 10^–01^	2 × 10^–01^
Example–2	Min	2 × 10^–08^	3 × 10^–08^	9 × 10^–08^	6 × 10^–08^	7 × 10^–08^	7 × 10–09	4 × 10^–08^	7 × 10^–08^	8 × 10–09	6 × 10^–08^
	Mean	4 × 10^–03^	4 × 10^–03^	4 × 10^–03^	4 × 10^–03^	4 × 10^–03^	4 × 10^–03^	4 × 10^–03^	5 × 10^–03^	5 × 10^–03^	5 × 10^–03^
	Max	2 × 10^–01^	2 × 10^–01^	2 × 10^–01^	2 × 10^–01^	2 × 10^–01^	2 × 10^–01^	2 × 10^–01^	2 × 10^–01^	2 × 10^–01^	2 × 10^–01^
	Med	4 × 10–05	7 × 10–05	1 × 10^–04^	1 × 10^–04^	1 × 10^–04^	2 × 10^–04^	2 × 10^–04^	2 × 10^–04^	2 × 10^–04^	2 × 10^–04^
	SIR	9 × 10–05	2 × 10^–04^	2 × 10^–04^	2 × 10^–04^	3 × 10^–04^	3 × 10^–04^	4 × 10^–04^	4 × 10^–04^	4 × 10^–04^	5 × 10^–04^
	STD	2 × 10^–02^	2 × 10^–02^	2 × 10^–02^	2 × 10^–02^	2 × 10^–02^	2 × 10^–02^	2 × 10^–02^	2 × 10^–02^	2 × 10^–02^	2 × 10^–02^
Example-3	Min	4 × 10^–08^	3 × 10^–08^	1 × 10^–07^	1 × 10^–07^	1 × 10^–07^	1 × 10^–07^	1 × 10^–07^	1 × 10^–07^	1 × 10^–07^	1 × 10^–07^
	Mean	1 × 10^–02^	1 × 10^–02^	1 × 10^–02^	1 × 10^–02^	1 × 10^–02^	1 × 10^–02^	1 × 10^–02^	1 × 10^–02^	1 × 10^–02^	1 × 10^–02^
	Max	6 × 10^–01^	6 × 10^–01^	6 × 10^–01^	7 × 10^–01^	7 × 10^–01^	7 × 10^–01^	7 × 10^–01^	7 × 10^–01^	7 × 10^–01^	7 × 10^–01^
	Med	5 × 10–05	1 × 10^–04^	1 × 10^–04^	1 × 10^–04^	1 × 10^–04^	1 × 10^–04^	2 × 10^–04^	2 × 10^–04^	2 × 10^–04^	2 × 10^–04^
	SIR	2 × 10^–04^	4 × 10^–04^	5 × 10^–04^	4 × 10^–04^	5 × 10^–04^	6 × 10^–04^	7 × 10^–04^	7 × 10^–04^	8 × 10^–04^	8 × 10^–04^
	STD	8 × 10^–02^	8 × 10^–02^	8 × 10^–02^	8 × 10^–02^	9 × 10^–02^	9 × 10^–02^	9 × 10^–02^	9 × 10^–02^	9 × 10^–02^	8 × 10^–02^

**Table 3:** **C**onvergence of the FMWNN-PSOSQP to solve the FBTMM

Examples	*FIT* ≤			*MSE* ≤			*TIC* ≤		
	10^–02^	10^–03^	*0*^–04^	*0*^–02^	10^–03^	10^–04^	10^–02^	10^–03^	10^–04^
1	58	56	51	58	54	30	69	65	61
2	59	57	52	59	55	43	68	61	58
3	59	58	50	59	53	41	69	62	60

Comparison of the outcomes of fractional MWNNs optimized with PSOASA for solving FBTMM has been made with reported results of state of the art deterministic and stochastic solver in order to access the performance rigorously. The absolute error of the reported numerical solver based on matric approach introduced by Podlubny [11], sigmoidal fractional neural networks optimized with IPA (FNN-IPA) [22, 23], sigmoidal neural networks optimized with GAs aided with pattern search (PS), i.e., GA-PS [54] and sigmoidal neural networks trained with particle swarm optimization (PSO) supported with PS, i.e., (PSO-PS) [20] are presented in Table 4 along with the proposed results of FMWNN-PSOASA. One can easily decipher from results presented in Table 4, the values of the AE for FMWNN-PSOASA are comparable to state-of-the-art deterministic and stochastic numerical procedures for solving FBTMM.

**Table 4:** Comparison of outcomes of FMWNN-PSOSQP with reported solution for the FBTMM in case of α = 1.5

*t*	AE of reference reported results					AE of Presented
	Numerical	GA-PS	PSO-PS	VIM	FNN-IPA	FMWNN-PSOASA
0.1	5.76 × 10^−−5^	3.43 × 10^−−2^	2.2 × 10^−−3^	5.48 × 10^−−5^	8.73 × 10^−−6^	2.14 × 10^–08^
0.2	8.29 × 10^−−5^	3.33 × 10^−−2^	2.63 × 10^−−3^	6.31 × 10^−−4^	1.12 × 10^−−5^	3.23 × 10^–08^
0.3	9.12 × 10^−−5^	3.04 × 10^−−2^	2.98 × 10^−−3^	2.66 × 10^−−3^	1.08 × 10^−−5^	9.38 × 10^–08^
0.4	8.74 × 10^−−5^	2.57 × 10^−−2^	2.97 × 10^−−3^	7.48 × 10^−−3^	8.19 × 10^−−6^	6.82 × 10^–08^
0.5	7.42 × 10^−−5^	1.96 × 10^−−2^	2.46 × 10^−−3^	1.67 × 10^−−2^	7.06 × 10^−−6^	7.19 × 10^–08^
0.6	5.36 × 10^−−5^	1.26 × 10^−−2^	1.49 × 10^−−3^	3.22 × 10^−−2^	1.01 × 10^−−5^	7.03 × 10^–09^
0.7	2.68 × 10^−−5^	5.49 × 10^−−3^	2.67 × 10^−−4^	5.8 × 10^−−2^	1.60 × 10^−−5^	4.60 × 10^–08^
0.8	5.07 × 10^−−5^	8.80 × 10^−−4^	8.34 × 10^−−4^	9.58 × 10^−−2^	2.03 × 10^−−5^	7.34 × 10^–08^
0.9	4.12 × 10^−−5^	5.42 × 10^−−3^	1.27 × 10^−−3^	1.5 × 10^−−1^	1.86 × 10^−−5^	8.92 × 10^–09^
1	8.08 × 10^−−5^	6.91 × 10^−−3^	3.05 × 10^−−4^	2.25 × 10^−−1^	1.24 × 10^−−5^	6.18 × 10^–08^

## Concluding remarks

The current work investigations are to design a neuro-swarming computational numerical procedure for the fractional Bagley–Torvik mathematical model. The optimization procedures based on the global search particle swarm optimization and local search active-set approach using the activation function Mayer wavelet neural network have been applied to solve the fractional model. The proposed stochastic solver MWNN-GAASA efficiency is performed to solve three different variants based on the fractional order of the FBTMM. For the exactness of the stochastic solver MWNN-PSOASA, the comparison of the attained and exact solutions will be provided for each variant of the FFBTMMM. The AE values have been obtained in good measures that are calculated around 10^−06^ to 10^−07^ for each example of the FBTMM. For the reliability of the proposed stochastic solver MWNN-PSOASA, the statistical soundings are provided based on the stability, robustness, accuracy, and convergence. One can conclude from these outcomes that around 75% independent trials achieved precise level of the accuracy. Beside the advantage of accurate and reliable outcomes of designed MWNN-PSOASA, the limitation of slowness of operation of global search with PSO and then local search with ASA.

## Further research openings

The FMWNN-PSOASA can be implemented to solve the fluid nonlinear models, fraction order systems, and fluid models [55–63]. Moreover, the used of heuristic methodologies having inherent strength of global as well as local search like differential evolution, backtracking search optimization algorithm, weights differential evolution, and their recently introduced variants are good alternative of integrated PSOASA.

## Data Availability

There is no data associated with this manuscript.
